# Individual Participant Data (IPD) Meta-analyses of Diagnostic and Prognostic Modeling Studies: Guidance on Their Use

**DOI:** 10.1371/journal.pmed.1001886

**Published:** 2015-10-13

**Authors:** Thomas P. A. Debray, Richard D. Riley, Maroeska M. Rovers, Johannes B. Reitsma, Karel G. M. Moons, Cochrane IPD Meta-analysis Methods group

**Affiliations:** 1 Julius Center for Health Sciences and Primary Care, University Medical Center Utrecht, Utrecht, The Netherlands; 2 The Dutch Cochrane Centre, Julius Center for Health Sciences and Primary Care, University Medical Center Utrecht, Utrecht, The Netherlands; 3 Research Institute for Primary Care and Health Sciences, Keele University, Staffordshire, The United Kingdom; 4 Radboud Institute for Health Sciences, Radboudumc Nijmegen, The Netherlands

**Summary Points**

- Individual participant data meta-analyses (IPD-MAs) provide unique opportunities not only for therapeutic studies but also for diagnostic and prognostic prediction modeling studies.
- IPD-MAs of prediction modeling studies allow for more robust development of prediction models, as well as for directly validating them and testing their generalizability across different (sub)populations and settings.
- Methods for IPD-MA of prediction modeling studies fundamentally differ from other types of IPD-MA research, because of the focus on the estimation of absolute risks and the importance of covariates.
- When heterogeneity is present in an IPD-MA of prediction models, special care is needed to enable tailoring of the prediction model to (sub)populations or settings at hand, to enhance their generalizability and usefulness.

## Introduction

A fundamental part of medical research is the development and validation of diagnostic and prognostic prediction models [1,2]. These prediction models aim to predict the absolute probability that a certain disease or condition is currently present (diagnostic models) or that an outcome will occur within a specific follow-up period (prognostic models) for an individual subject.

Prediction models typically rely on multiple predictors, which can include demographic characteristics, medical history and physical examination items, or more complex measurements from, for example, medical imaging, electrophysiology, pathology, and biomarkers. Also for diagnostic models, estimates of probabilities are rarely based on a single test, and doctors naturally integrate several patient characteristics and symptoms [3]. A broad range of prediction modeling techniques exist, like regression approaches, neural network models, decision tree models, genetic programming models, and support vector machine learning models, although prediction models developed by a multivariable regression approach are by far prevailing.

**Fig 1:**
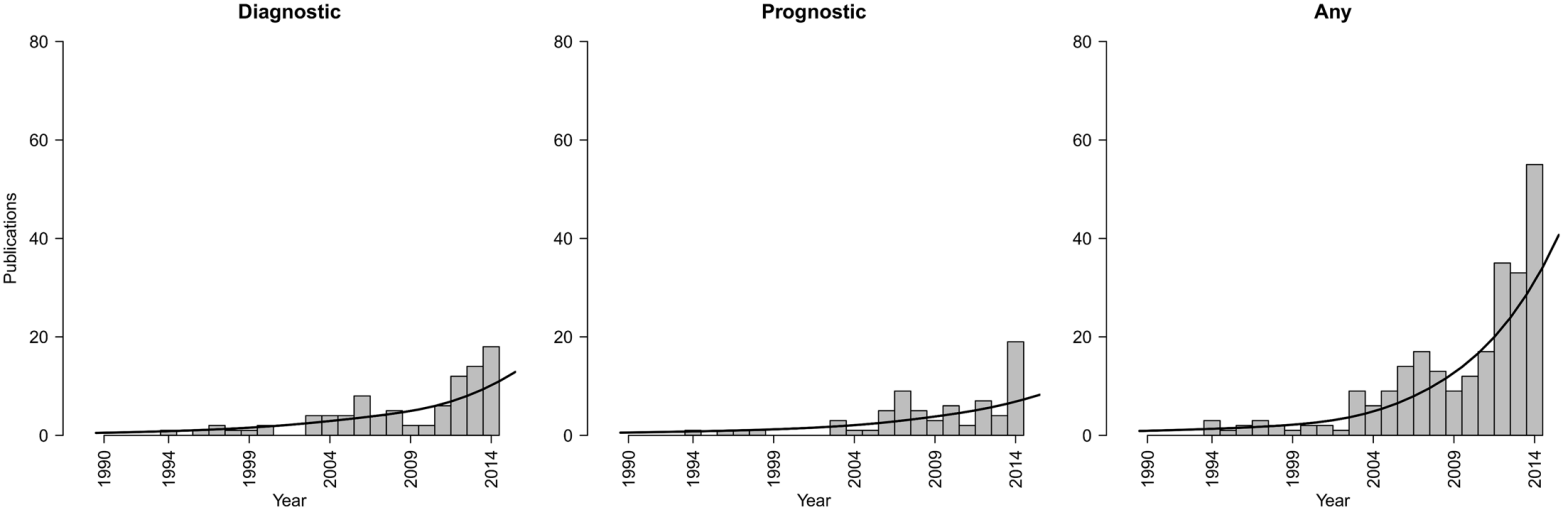
Trends in publications of IPD-MA studies focusing on the development and/or validation of diagnostic or prognostic prediction models. Number of publications per year focusing on diagnostic, prognostic, or either type of IPD-MA. Results were identified by applying the search strategy of Riley et al. [14] in PubMed on March 24, 2015. A sensitive filter was applied to identify those publications explicitly mentioning the study aim (diagnosis, prognosis, or prediction) in the title.

It is widely recommended that a developed prediction model should not be used in practice before being externally validated—at least once—in other individuals than those used for model development [4–7]. Unfortunately, most prediction models are poorly or not at all validated, rendering interpretation of their generalizability difficult. In addition, many systematic reviews showed that for the same outcome or same target population, numerous competing models exist [8–10]. Generally speaking, researchers often ignore existing prediction models and develop yet another prediction model from their own data [2]. This practice sustains a cycle of underpowered prediction model development studies and poor knowledge about the generalizability and applicability of developed prediction models. Evidence synthesis and meta-analysis of individual participant data (IPD) from multiple studies seems to be a unique opportunity to address these problems, as it allows researchers to develop and directly validate models on large datasets and across a wide range of populations and settings, to directly test a model’s generalizability (Fig 1) [11–13].

There is currently little guidance on how to conduct an IPD meta-analysis (IPD-MA) for developing and/or validating diagnostic or prognostic prediction models [15]. To date, most IPD-MA articles focus on estimating relative quantities, like a risk ratio, hazard ratio, or odds ratio for a specific treatment or a specific etiologic factor. In contrast, prediction modeling research is focused on developing and validating multivariable models aimed at calculating an absolute risk estimate of the combined variables, rather than estimating the relative effect of a specific treatment or etiologic factor. Furthermore, prediction modeling studies focus entirely on the role and joint contribution of multiple covariates, whereas intervention studies in principle rely on randomization to reduce the role of covariates (Table 1). Hence, IPD-MAs of randomized intervention and etiological studies, which are beyond the scope of this paper and are instead addressed in the accompanying paper [16], differ from IPD-MAs of multivariable prediction models, which are the focus of this paper.

**Table 1:** The main differences between IPD-MA of treatment intervention studies and of multivariable prediction modeling studies.

	Intervention Research	Diagnostic/Prognostic Modeling Research
**General Issues**		
Primary aim	Estimation of therapeutic effect of a specific treatment	Estimation of the probability of the presence (diagnosis) or future occurrence (prognosis) based on combinations of two or more predictors
Secondary aims	Treatment effect in study subgroups	Evaluate accuracy of model predictions across subgroups, settings, or countries
Estimates of interest	(Adjusted) treatment-outcome associations	(Distribution of) individual outcome probabilities/risks; discrimination and calibration of estimated model probabilities
Association measures	Relative risk estimates: risk ratio, hazard ratio, risk difference, and odds ratio	Absolute probability or risk estimates of the outcome at interest
Study design	Randomized studies	Observational research (randomized study data sometimes also used)
**Data retrieval**		
Study registration	Clinical Trials registry (http://clinicaltrials.gov/)	No such registry
Developing search query	Extensive Cochrane Collaboration guidance, including search filters	Recent but less evolved guidance (by Cochrane Collaboration)
Assessing risk of bias	Risk of Bias tool (Cochrane Collaboration)	CHARMS tool (Cochrane Collaboration)
**Data analysis**		
Statistical model	Models yielding valid estimates of relative treatment effects	Models yielding absolute outcome probabilities
Relevance of covariates	Covariates may be included to adjust for baseline imbalance and to investigate potential effect modifiers	Covariates (other predictors) are explicitly included to increase the model’s predictive accuracy
Dealing with between-study heterogeneity	Random-effects modeling of treatment effect, inclusion of treatment-covariate interactions, meta-regression, and subgroup analysis	Stratification of baseline risk across studies, focus on homogeneous and weakly heterogeneous predictors, and inclusion of interaction terms and nonlinear predictor effects
Validation of research findings	Comparison of model fit, sensitivity analyses, and recursive cumulative meta-analysis	Evaluation of model discrimination and calibration; internal, internal-external, and external validation
Measures of precision	Standard error, *p*-value, confidence intervals of (relative) treatment effect, and prediction intervals	Confidence and prediction intervals of model discrimination and calibration

Abbreviations: CHARMS, Checklist for Critical Appraisal and Data Extraction for Systematic Reviews of Prediction Modeling Studies

We provide an overview of the advantages and limitations of IPD-MAs aiming to develop a novel prediction model or to validate one or more existing models across multiple datasets. This overview is based on published guidelines and existing recommendations for the conduct of prediction modeling studies and of IPD-MA research. We illustrate this overview with examples of recently published IPD-MAs of prediction models across various medical domains. Our aim is to help researchers, readers, reviewers, and editors to identify and understand the key issues involved with such IPD-MA projects.

## Types of IPD-MA of Prediction Modeling Studies

Several types of IPD-MA can be distinguished. When a single (previously published) prediction model is available, typical research aims include the following:

- To validate and summarize the model’s performance across various study populations, settings, and domains. For example, Geersing and colleagues used an IPD-MA to examine the predictive accuracy of the Wells rule for diagnosing deep vein thrombosis (DVT) across different subgroups of suspected patients [17].
- To tailor (update) the model to specific populations or settings. For instance, Majed and colleagues evaluated whether the calibration of the Framingham risk equation for coronary heart disease and stroke improved by applying local adjustments [18].
- To examine the added value of a specific predictor or (bio)marker to the model across different study populations, settings, and domains. For example, an IPD-MA was performed to summarize the added value of common carotid intima-media thickness (CIMT) in 10-year risk prediction of first-time myocardial infarctions or strokes in the general population, above that of the Framingham Risk Score [19].

When various competing prediction models are available that were developed for the same target population or the same outcome across various study populations, typical research aims of an IPD-MA include the following:

- To compare the models’ performance across various study populations, settings, and domains. For instance, an IPD-MA was used to validate and compare all noninvasive risk scores for the prediction of developing type 2 diabetes in individuals of the general population [20].
- To combine the most promising models and adjust them to specific study populations, settings, and domains. This approach is illustrated by Debray and colleagues, who validated and updated all existing diagnostic models for predicting the presence of DVT across different settings and proceeded to combine them into a single meta-model [21].

Finally, when no prediction models are available, an IPD-MA can be used to develop and directly validate a new prediction model using the IPD from all relevant studies. An example is the development of the prognostic PHASES score for prediction of risk of rupture of intracranial aneurysms in patients with aneurysms but without any treatment [22].

## Advantages and Challenges of an IPD-MA in Prediction Research

Box 1 summarizes the general advantages and challenges of IPD-MA in prediction studies. An IPD-MA of prediction models has a major advantage not only in developing more robust prediction models because of the increased sample size but also in directly validating the models and hence revealing their clinical usefulness. This combination of development, validation, and testing for usefulness can be applied across different patient subgroups, different target populations, and even different care settings, if such datasets are included in the IPD-MA. Whereas a single prediction modelling study is usually confined to quantifying the average predictive performance of the prediction model across the entire study population, an IPD-MA allows for quantifying subgroup- or setting-specific performances, and the prediction model can even be tailored to optimize its performance in these specific (sub)groups or settings. For example, Geersing and colleagues examined the diagnostic accuracy of the Wells prediction rule for diagnosing DVT across different subgroups of suspected patients and evaluated whether the rule had to be tailored to enhance its accuracy in these subgroups [17]. Table 2 highlights unique advantages of IPD access for different research aims.

**Box 1. Advantages and Challenges of IPD-MA of Multivariable Prediction Modeling Studies, Based on Ahmed and colleagues [15]**

**Advantages**

- Increases the total sample size. This reduces the risk of incidental findings, increases the precision of study results, and enables the development of more robust prediction models.
- Increases the available case-mix variability. This enhances the potential generalizability of prediction models across subgroups, settings, and countries.
- Ability to standardize analysis methods across IPD sets. For example, one can standardize the type of statistical model used (such as Cox, logistic or other model), the predictor and outcome definitions, and the methods to account for differences in censoring and in lengths of follow-up.
- Ability to investigate more complex associations, such as nonlinearity of predictor effects, predictor interactions, and time-varying predictor effects.
- Ability to explore heterogeneity in the predictive performance of the models—for example, in whom (in which subgroups, countries, or settings) or under which circumstances does a prediction model not perform adequately.
- Ability to evaluate generalizability and thus usability of prediction models across different situations.

**Challenges**

- Unavailability of IPD in some studies and assessing the impact of their absence on predictive performance of a developed or validated prediction model.
- Methodological quality assessment of primary prediction modeling studies is yet less well developed.
- Dealing with different definitions of predictors and outcomes; with different data sources (such as prospective and retrospective cohort studies, case-control studies, case-cohort studies, or randomized trials); and with different (or outdated) treatment strategies, especially when older and newer primary studies are combined.
- Dealing with missing data, including partially missing predictors and outcome data, as well as completely missing predictors in some studies.
- Dealing with heterogeneity in predictor effects and outcome occurrence across the included primary studies.

**Table 2:** Overview of types (aims) of IPD-MAs of prediction modeling studies.

Starting Point	Use IPD Datasets to	Apply Meta-analysis to	What Aggregate Data Can Be Used?	IPD Access Allows to
Existing prediction model(s)	Validate these models.	Pool estimates of model discrimination and calibration.	Published estimates of the predictive performance	Investigate sources of heterogeneity in model performance; identify which models perform best in what (sub)population, setting, or country.
Existing prediction model(s)	Tailor (update) or combine these models.	Adjust for between-study heterogeneity in outcome occurrence and/or predictor effects.	Published prediction models and published predictor effects	Combine and tailor the model(s) to specific (sub)populations, settings, or countries.
Existing prediction model(s)	Investigate added value of new predictor(s) to existing model.	Pool estimates of added value (such as adjusted predictor effect or improvement in model calibration, discrimination, and/or reclassification).	Published estimates of added value of specific predictor to a specific model	Investigate sources of heterogeneity in added value; identify relevant subgroups that yield different added value.
No existing prediction model(s)	Develop new model.	Adjust for between-study heterogeneity in outcome occurrence or predictor effects.	Published predictor effects	Tailor the meta-model to specific (sub)populations, settings, or countries.

Ahmed and colleagues recently provided over 20 recommendations to improve the development and validation of prediction models using IPD from multiple studies [15]. We here elaborate on five key aspects of IPD-MA of prediction models: prespecifying the IPD-MA, identifying the relevant studies for the IPD-MA, assessment of risk of bias of individual studies/datasets, implementation of appropriate statistical methods, and reporting of results.

## Prespecifying the IPD-MA

It is important for IPD-MA projects that one a priori defines the rationale, methods, conduct, and analyses of the IPD-MA [23]. When IPD-MA projects are based on a systematic review, the protocol should also indicate which type of publications are deemed relevant. Researchers may, for instance, seek all publications that have already developed or validated a prediction model for a specific target population, specific setting, or for specific outcome(s) [24–26]. Alternatively, researchers may seek all publications that used a dataset that fit the IPD-MA objective. Study protocols are certainly relevant for IPD-MAs that are prospectively planned and should ideally be accessible for inspection by external parties [23,27,28]. By setting common quality standards and standardizing predictor definitions, measurement methods, and outcome recoding, the consistency across included datasets increases, thereby reducing the risk of bias. Study protocols may also help to convince other researchers to participate in the IPD-MA and to share their IPD. Examples of relevant databases for protocols of systematic reviews are the Cochrane Library (www.thecochranelibrary.com) and the International Prospective Register of Systematic Reviews (www.crd.york.ac.uk/prospero/).

## Identifying the Relevant Studies for the IPD-MA

Various competing strategies can be initiated for collecting the relevant studies in an IPD-MA [15]. Similar to meta-analyses of randomized trials of treatments [16,29,30], these studies should ideally be identified through a systematic review. However, it is also possible to prospectively set up a collaborative group of selected researchers active on the same topic who agree to share their IPD. Examples of such collaborations are the European Prospective Investigation into Cancer and Nutrition (*N* > 520,000 from ten European countries) [31] and the Emerging Risk Factors Collaboration (*N* > 2.2 million participants in 125 prospective studies) [32]. Furthermore, if there are known relevant studies that did not provide their IPD, it may help to extract their summary information. Examples of such information are measures of model performance including c-statistic and calibration slopes with their 95% confidence intervals and estimated predictor effects. Recently, methods have been developed to combine such summary estimates with the results from the IPD-MA [21,33,34].

## Assessing Risk of Bias of Included Studies

Similar to any type of systematic review, the quality of an IPD-MA of prediction models strongly depends upon the methodological quality of included studies. When these studies have flaws in the design, conduct, or analysis, the IPD-MA may yield biased estimates of predictive performance. Researchers should therefore evaluate the methodological quality in each of the included prediction model studies. The CHARMS checklist is a very recent guideline and checklist for data extraction and critical appraisal of primary prediction model studies in systematic reviews [35]. This checklist may also be used for critically appraising the primary prediction modeling studies to be included in the IPD-MA. Progress on a formal risk-of-bias tool for prediction modeling studies, developed by various authors around the globe, including co-conveners of the Cochrane Prognosis Methods Group, is underway (www.systematic-reviews.com/probast/).

## Statistical Methods

The statistical analysis approach of an IPD-MA of prediction models has to deal with several key issues. We here elaborate on missing data and between-study heterogeneity. Another key issue is the combination of IPD and aggregate data from the literature (Table 2). Combining IPD and aggregate data for the development and validation of multivariable prediction models is, however, not straightforward and therefore beyond the scope of this paper. Statistical methods for this purpose have previously been described [21,33,34,36].

### Missing Data

Missing data basically appear when subject characteristics have not fully been recorded within the primary studies or were measured inconsistently across studies (for example, a predictor that was measured on a continuous versus categorical scale), so-called partial missing data. Missing data may also arise when some of the included studies did not measure a certain predictor at all, the so-called systematically missing predictors [37–39]. In any case, it is increasingly acknowledged that missing data should be addressed using multiple imputation techniques; this should also be done in IPD-MAs. These techniques generate (multiple) completed versions of each original dataset. For partial missing data, imputation can be applied separately for each study of the IPD-MA to allow for heterogeneity in associations between observed and missing predictors. However, when there is a mixture of partial and systematically missing data, more advanced imputation techniques are needed to simultaneously impute the IPD from each study in the IPD-MA [37,38].

### Between-Study Heterogeneity

Between-study heterogeneity arises when the studies of an IPD-MA yield substantially different estimates of model performance (for example, when validating an existing prediction model), of a predictor’s added predictive value (for example, when examining the added value of a certain predictor to an existing prediction model), or of certain predictor effects (for example, when developing a novel prediction model). Researchers should therefore investigate the presence of heterogeneity across the available studies and check whether its extent can be reduced or explained. As a first step, researchers may compare the characteristics of included studies and populations. Depending on the research aim of the IPD-MA, different methods can then be applied to account for the presence of between-study heterogeneity.

When externally validating one or more prediction models in an IPD-MA, it is recommended to investigate the influence of specific study characteristics such as case-mix differences on the predictive performance of the models [40,41]. This could reveal under which circumstances the model remains valid and how the model may be improved upon for different subgroups or settings. The presence of between-study heterogeneity in model performance can be investigated using traditional (random effects) techniques that are sometimes used in the analysis of multinational or multicenter randomized trials. For instance, Kengne and colleagues validated all existing models for predicting the development of type 2 diabetes in the general population, separately in different countries, and then pooled the resulting performance estimates using a traditional random effects model [20]. When such random effects analysis conveys substantial heterogeneity in the performance of a prediction model in a certain country, (sub)population, or setting, an IPD-MA allows one to tailor or update the prediction model to enhance its performance in that specific country, subpopulation, or setting. An example is the validation and updating of the sex-specific Framingham risk equation for coronary heart disease and stroke [18]. An IPD-MA was used to adjust this model for the baseline survival and mean predictor values of each included country and to re-estimate country-specific predictor effects. The study concluded that the updated model, despite yielding poor discrimination, achieved better calibration in a European population of middle-aged men.

When investigating the added value of a specific (new) predictor to existing predictors, it is recommended to verify whether this added predictive value substantially varies across the included studies of an IPD-MA and, if so, to evaluate under which circumstances and in which types of individuals or settings it can be used as an addition to existing predictors or models. For example, Den Ruijter and colleagues tested heterogeneity in the added value of CIMT above that of the Framingham Risk Score by exploring the presence of interaction between cohort membership and CIMT [19]. Because no evidence for heterogeneity was found and because the improvement in 10-year risk prediction of first-time myocardial infarction or stroke was small, they concluded that the addition of CIMT on top of Framingham Risk Score is unlikely to be of clinical importance.

When developing a novel prediction model from an IPD-MA, researchers may quantify the degree of variation in outcome frequency and in predictor effects across the IPD-MA studies using traditional meta-analysis methods [11]. This information could then be used to decide which and how predictors will be included during the statistical analyses. It has been demonstrated that using average predictor effects (for instance, as obtained from random effects models) is detrimental when their association substantially varies across studies [11]. For this reason, efforts should be made to facilitate tailoring of the model to new study populations. This can, for instance, be achieved by applying stratification, by omitting predictors with heterogeneous effects, or by considering nonlinear terms and interaction effects. In contrast to prediction models developed from a single dataset, an IPD-MA has the unique feature of directly applying internal-external cross validation of a developed model [11,13,42]. This method iteratively discards one study of the IPD-MA for external validation purposes and uses the remaining studies for the model development. This directly allows one to investigate whether a developed model predicts differently across certain populations, settings, or even subgroups and whether the model would differ in fit for purpose when applied in practice. Also, it is an ideal method to deal with between-study heterogeneity to determine the extent of the generalizability and thus applicability of the developed model. Finally, when the calibration and discrimination performance measures from an internal-external cross validation are pooled using a multivariate meta-analysis approach [40], it is even possible to evaluate whether a prediction model’s average performance varies across different subgroups, populations, or settings. This in turn helps to identify under what circumstances in clinical practice the model can reliably be used and when and whether a developed model first requires tailoring to specific clinical situations to enhance its usefulness for the situation at hand.

## Reporting IPD-MA of Prediction Modeling Studies

Similar to meta-analysis of randomized trials of interventions [43], IPD-MA should be reported fully and transparently as well, to allow readers to assess the strengths and weaknesses of the investigation. Although specific guidelines are currently lacking, important issues to report should clearly include details on study identification, study inclusion and exclusion criteria, predictor and outcome definitions, the amount of missing subject-level and missing study-level data, the presence of between-study heterogeneity, and how this is dealt with [15]. Furthermore, to address potential concerns over selective nonpublication, authors should explain whether analyses could be completed as planned or why they had to be revised. Further—but less specific—guidance can be found in the recent Preferred Reporting Items for Systematic Reviews and Meta-Analyses (PRISMA)-IPD reporting statement [44] and in the very recent Transparent Reporting of a Multivariable Prediction Model for Individual Prognosis or Diagnosis (TRIPOD) reporting guideline for studies on developing, validating, or updating a prediction model [3,45].

## Conclusions

Systematic review and meta-analysis of IPD is widely recognized as a gold standard approach in intervention research and is equally pivotal in prediction modeling research. Having IPD from multiple studies is particularly useful for improving the performance of novel prediction models across different study populations, settings, and domains and to attain a better understanding of the generalizability of prediction models. It is, however, important to acknowledge that IPD-MAs are no panacea against poorly conceived and conducted primary studies. Well-designed prospective studies remain paramount and could, for instance, benefit by involving multiple centers or countries and applying IPD-MA methodology. Prospective studies are also needed for evaluating the impact of prediction models on decision making and patient outcomes. The guidance provided in this article may help researchers to decide upon appropriate strategies when conducting an IPD-MA in prediction modeling research and assist readers, reviewers, and practitioners when evaluating the quality of resulting evidence.

## Abbreviations

CIMT: carotid intima-media thickness
CHARMS: Checklist for Critical Appraisal and Data Extraction for Systematic Reviews of Prediction Modeling Studies
DVT: deep vein thrombosis
IPD: individual participant data
MA: meta-analysis
PRISMA: Preferred Reporting Items for Systematic Reviews and Meta-Analyses
TRIPOD: Transparent Reporting of a Multivariable Prediction Model for Individual Prognosis or Diagnosis
